# Suppression of CLC-3 chloride channel reduces the aggressiveness of glioma through inhibiting nuclear factor-κB pathway

**DOI:** 10.18632/oncotarget.19093

**Published:** 2017-07-08

**Authors:** Bing Wang, Jing Xie, Hai-Yong He, En-Wen Huang, Qing-Hua Cao, Lun Luo, Yong-Shi Liao, Ying Guo

**Affiliations:** ^1^ Department of Neurosurgery, The Third Affiliated Hospital of Sun Yat-Sen University, Guangzhou, China; ^2^ Department of Neurosurgery, The Second Affiliated Hospital, University of South China, Hengyang, China; ^3^ Department of Integrative Oncology, Shanghai Cancer Center, Fudan University, Shanghai, China; ^4^ Faculty of Forensic Medicine, Zhongshan School of Medicine, Sun Yat-Sen University, Guangzhou, China; ^5^ Department of Pathology, The First Affiliated Hospital of Sun Yat-Sen University, Guangzhou, China

**Keywords:** CLC-3, glioma, invasion, nuclear factor-κB, matrix metalloproteinase

## Abstract

CLC-3 chloride channel plays important roles on cell volume regulation, proliferation and migration in normal and cancer cells. Recent growing evidence supports a critical role of CLC-3 in glioma metastasis, however, the mechanism underlying is unclear. This study finds that CLC-3 is upregulated in glioma tissues and positively correlated with WHO histological grade. Patients with high CLC-3 expression had an overall shorter survival time, whereas patients with low expression of CLC-3 had a better survival time. Silencing endogenous CLC-3 with ShCLC-3 adenovirus significantly decreases volume-regulated chloride currents, inhibits the nuclear translocation of p65 subunit of Nuclear Factor-κB (NF-κB), decreases transcriptional activity of NF-κB, reduces MMP-3 and MMP-9 expression and decreases glioma cell migration and invasion. Taken together, these results suggest CLC-3 promotes the aggressiveness of glioma at least in part through nuclear factor-κB pathway, and might be a novel prognostic biomarker and therapeutic target for glioma.

## INTRODUCTION

Glioma is the most common and a highly aggressive primary malignant tumor in the central nervous system [1]. Even with aggressive treatment with surgery, radiation and chemotherapy, the prognosis for patients with malignant gliomas remains poor. The cumulative 1-year survival rate of glioma patients is less than 30%, and median reported survival of glioblastoma is 12-15 month [2]. The dismal prognosis of glioma patients is largely attributed to the highly invasive nature of glioma cells, which makes complete surgical resection of gliomas extremely difficult. The molecular determinants of disease aggressiveness are not well understood. A better understanding of the molecular pathogenesis of glioma may identify new targets for treatment of this typically incurable brain cancer.

A salient feature of glioma aggressiveness is, instead of passive metastasis through vascular circulation, the malignant cells actively protrude along the brain vasculature, diffusively infiltrate the adjacent normal tissue without clearly delineated borders [3]. Invasion within the spatial constraints of the mature brain require special adaptations for these invading cells. For example, glioma cells need to undergo shape changes and volumetric alterations as they squeeze through narrow extracellular brain spaces. Critical to these volume modifications is the ion channels that regulate the flux of ions in and out of the cell, followed by osmotically obligated water [4]. Cl^−^ is the primary anion in both extracellular fluid and intracellular medium. Chloride channels have been drawing increasing interests in cancer biology [5]. We previously found that Ano1, a calcium-activated chloride channel is overexpressed in gastric cancer and promotes gastric cancer cell invasiveness by regulating epithelial-to-mesenchymal transition [6]. Recently growing evidence suggests CLC-3, one member of CLC voltage-gated chloride channel family, may play an important role in cell proliferation, migration and invasion in various types of cancer [5]. Overexpression of BCL-2 in human prostate cancer epithelial cells significantly increased chloride currents which were suppressed by CLC-3-specific antibody [7]. CLC-3 knockdown significantly inhibited migration and invasion in human glioma cells [8, 9] and other cancer cells [5].

Cumulating evidence support a crucial role of CLC-3 in cancer metastasis, however, the molecular mechanisms underlying are currently poorly understood. Recent growing evidence demonstrated that Nuclear Factor-κB (NF-κB)-mediated signaling pathway is constitutively activated in glioma cell lines and gliomas. Inhibition of NF-κB activation inhibits the proliferation and invasiveness of glioma cells [10, 11]. Whether NF-κB is involved in the oncogenic role of CLC-3 remains unknown. Interestingly, it was found several cytokines [12], including tumor necrosis factor-α [13, 14] and interleukin 1β [15], which are well-defined NF-κB activators, could activate chloride currents which are dependent on CLC-3 expression.

In the present study we set out to examine the expression of CLC-3 in clinical glioma samples, and investigated whether depletion of endogenous CLC-3 could inhibit cell migration and invasion and modulate the NF-κB transactivation in glioma cells.

## RESULTS

### CLC-3 is overexpressed in glioma tissues

**Figure 1:**
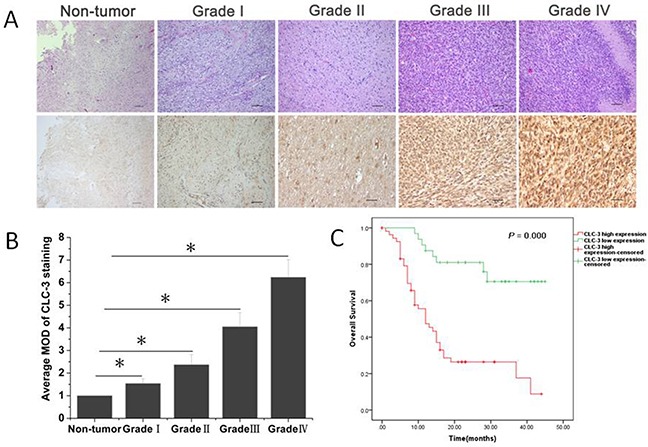
CLC-3 protein was overexpressed in glioma histopathological sections **(A)** Representative images of hematoxylin and eosin (HE) staining and immunohistochemistry (IHC) analysis of glioma sections and normal brain tissue. **(B)** The average MOD of CLC-3 staining increased as the glioma progressed to more advanced stages. *p < 0.05. n=6-35. **(C)** Survival curve for 89 glioma patients according to CLC-3 protein expression status (log-rank test).

Recently growing evidence supported functional roles of CLC-3 chloride channels on glioma cell cycle transition, proliferation and invasion [9, 12, 16, 17], however, the expression of CLC-3 protein in glioma tissues were poorly understood. To examine the expression of CLC-3 across glioma patients, immunohistochemistry assay on primary tumor specimens of four different grades of gliomas from 89 patients were performed. The results showed that CLC-3 expression was up regulated in glioma and its overexpression was positively correlated with WHO histological grade (Figure 1A, 1B, Supplementary Table 2). Furthermore, A Kaplan–Meier analysis and the log-rank test showed that the survival time between the low and high level of CLC-3 expression groups was significantly different(n = 89; p < 0.05, Figure 1C, Table 1). Patients with high CLC-3 expression had an overall shorter survival time, whereas patients with low expression of CLC-3 had a better survival time. There was a significant impact of WHO grade, a well known clinicopathological prognostic parameter on patients’ overall survivial. However, there was no significant correlation between overall survivial and the other clinicopathological parameters, such as gender, age at surgery (Table 1).

**Table 1:** Univariate and multivariate analysis of different prognostic factors for overall survival of patients with gilomas

Variable	All cases	Univariate analysis		Multivariate analysis		
		Mean ± SE^a^	*P* value^b^	HR^c^	95% CI^d^	*P* value
Gender						
Male	62	26.78±2.32	0.287	2.153	1.064-4.355	0.540
Female	27	21.99±3.19				
Age at surgery						
<35^e^	47	23.84±2.22	0.329	1.129	0.523-2.438	0.758
≥35	42	28.04±3.45				
WHO grade						
I	9	44.33±0.61	**0.000**	3.710	2.374-5.797	**0.000**
II	25	39.24±2.66				
III	20	18.74±2.15				
IV	35	12.50±2.08				
CLC-3 protein expression						
High	57	17.82±2.07	**0.000**	0.170	0.072-0.402	**0.000**
Low	32	36.98±2.45				

^a^SE, standard error; ^b^chi-square test; ^c^HR, hazard ratio; ^d^CI, confience interval; ^e^median age

### Knockdown of CLC-3 reduced migration and invasion of glioma cells

**Figure 2:**
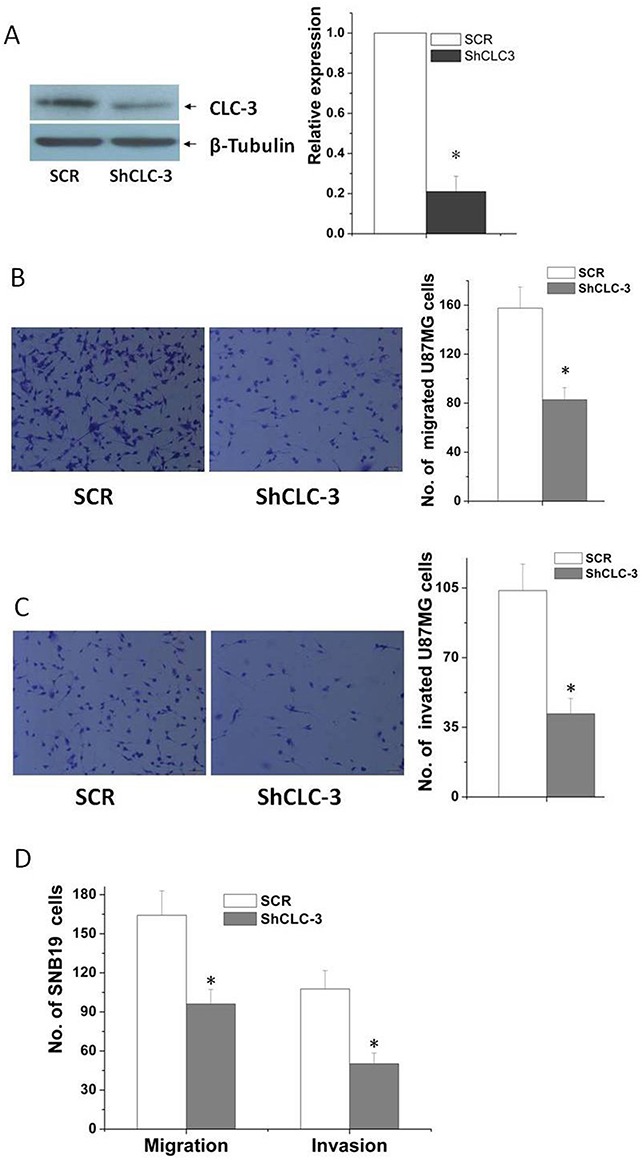
Knockdown of CLC-3 inhibited migration and invasion of glioma cells **(A)** Knockdown of CLC-3 in glioma cells was analyzed by western blotting using an anti- CLC-3 antibody. β-Tubulin was used as a loading control. **(B)** In the transwell assay, migrated U87MG cells significantly reduced after CLC-3 knockdown (*n* = 6, *p < 0.05 vs. CTR group). **(C)** In the transwell invasion assay, invasiveness was quantified by U87MG cells through Matrigel and it was showed fewer cells in the ShCLC-3 group than the SCR group (*n* = 6, *p < 0.05 vs. SCR group). **(D)** Knockdown of CLC-3 significantly inhibited migration and invasion of SNB19 cells (*n* = 6, *p < 0.05 vs. SCR group).

CLC-3 was found to play important roles on cell volume regulation, cell migration and invasion in normal [18, 19] and cancer cells [5]. CLC-3channels were upregulated in human glioma cell membranes [20]. Inhibition of CLC-3 channels by pharmacological inhibitors or CLC-3 siRNA transfection suppressed the invasion of human glioma cells [8, 9]. Here we investigated functional roles of CLC-3 in glioma cell migration and invasion by using recombinant adenovirus expressing short hairpin RNA targeted human *clc-3* gene. ShCLC-3 adenovirus significantly reduced endogenous CLC-3 protein expression (Figure 2A). A MTT assay showed that knockdown of CLC-3 did not significantly change the growth rate of glioma cells at 20 h (Supplementary Figure 1), however, knockdown of CLC-3 significantly inhibited the migration of U87MG cells (Figure 2B). Furthermore, a transwell matrix penetration assay (with Matrigel) showed that knockdown of CLC-3 by ShCLC-3 adenovirus profoundly repressed the invasive ability of U87MG cells (Figure 2C). Similar results were found in the SNB19 cells (Figure 2D).

### Knockdown of CLC-3 inhibited volume-regulated chloride currents

**Figure 3:**
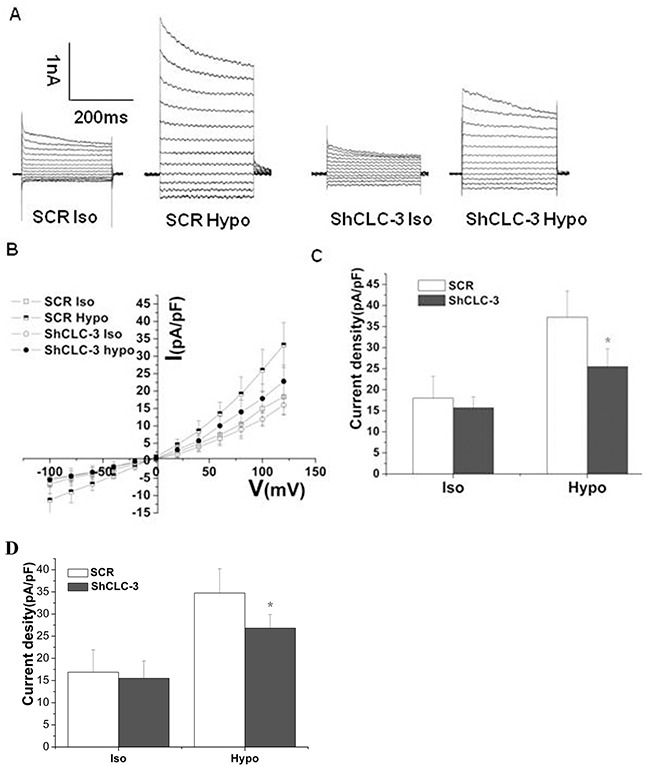
Knockdown of CLC-3 reduced volume-regulated chloride currents Representative traces **(A)**, I-V curves **(B)** and mean current densities (**C**) of Cl^−^ currents in isotonic and hypotonic solution in U87MG cells (n=7, #p<0.05 *vs*. SCR group). **(D)** Mean current densities of Cl^−^ currents in isotonic and hypotonic solution in SNB19 cells (*n* = 6, *p < 0.05 vs. SCR group). Iso, isotonic solution; Hypo, hypotonic solution.

Studies in normal [21] and cancer cells [5] support CLC-3 function as a key component or regulator of the volume-activated chloride channel. Hypotonic solution evoked a native volume-regulated Cl^−^ current (I_Cl.vol_), with a decrease in cytoplasma Cl^−^ concentration ([Cl^−^]_i_). CLC-3 antisense transfection or CLC-3-specific antibody reduced I_Cl.vol_ and Cl^−^ efflux induced by hypotonic challenge [7, 22]. We found that ShCLC-3 adenovirus significantly reduced volume-regulated chloride currents in U87MG and SNB19 cells (Figure 3).

### Knockdown of CLC-3 inhibited the transcriptional activity of NF-κB

**Figure 4:**
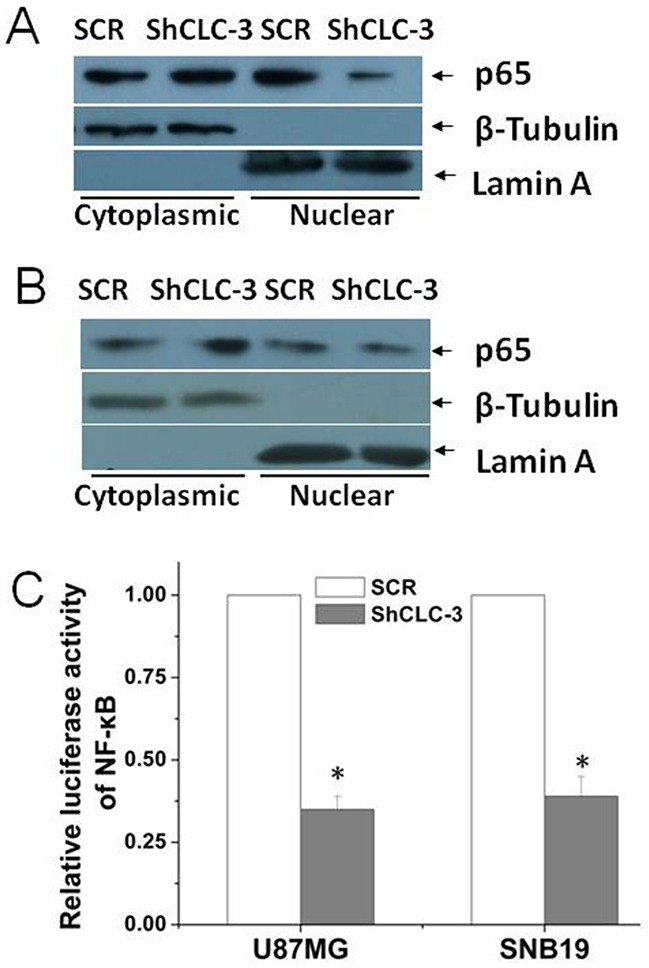
Knockdown of CLC-3 inhibited the transcriptional activity of NF-κB **(A)** Knockdown of CLC-3 inhibited nuclear localization of p65 in U87MG cells. Lamin A was used as a nuclear protein marker, and β-tubulin was used as a loading control. **(B)** Knockdown of CLC-3 inhibited nuclear localization of p65 in SNB19 cells. **(C)** Knockdown of CLC-3 significantly decreased the transcriptional activity of NF-κB as indicated by the luciferase reporter activity assay in U87MG and SNB19 cells (*n* = 6, *p < 0.05 vs. SCR group).

Recent studies found that a deficiency of CLC-3 led to a diminished activity of NF-κB in vascular smooth muscle cells and endothelial cells [14, 15], however, the mechanism of how CLC-3 regulates NF-κB signaling remained elusive. In U87MG and SNB19 cells it was found here that knockdown of CLC-3 significantly reduced p65 nuclear translocation (Figure 4A, 4B). As nuclear accumulation of p65 subunit of NF-κB was required for NF-κB transcriptional activation, we further investigated whether silencing CLC-3 altered the transcriptional activity of NF-κB. As shown in Figure 4C, a NF-κB reporter luciferase activity assay showed that knockdown of CLC-3 significantly inhibited the NF-κB transactivity in U87MG and SNB19 cells. A NF-κB reporter luciferase activity assay in LN229 and U373MG cells found similar results (Supplementary Figure 2).

### Knockdown of CLC-3 repressed MMP-3/9 expression

**Figure 5:**
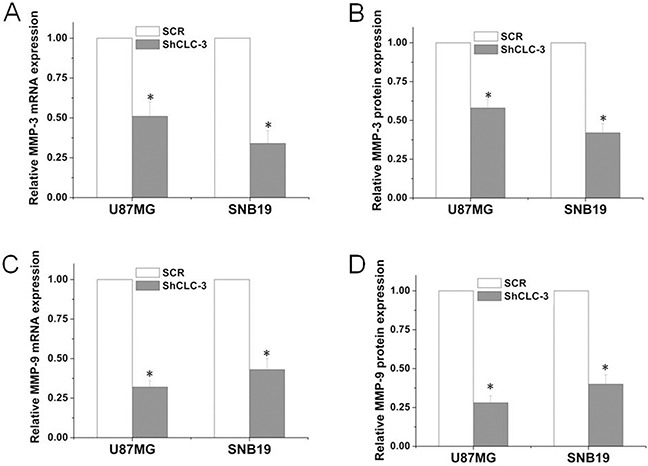
Knockdown of CLC-3 inhibited MMP-3 and MMP-9 expression Quantification of changes in *MMP-3* **(A)** and *MMP-9* **(C)** mRNA levels U87MG and SNB19 cells from ShCLC-3 group and SCR group. Expression levels of mRNA are presented as increasing fold compared with the control cells and were normalized with β-actin (*n* = 6, *p < 0.05 vs. SCR group). MMP-3 **(B)** and MMP-9 **(D)** protein levels in the supernatants of the indicated cells were assessed using ELISA. (*n* = 6, *p < 0.05 vs. SCR group).

NF-κB can transcriptionally up-regulate matrix metalloproteinases, such as MMP-3 and MMP-9. Increased expression of MMP-3/9 has been associated with glioma progression [10, 23]. We then investigated whether silencing CLC-3 altered the expression of MMP-3/9. Real-time PCR analysis revealed that the knockdown of CLC-3 drastically repressed MMP-3 expression at the mRNA level in U87MG and SNB19 cells (Figure 5A). Consistent with the PCR results, an ELISA assay of MMP-3 further demonstrated that knockdown of CLC-3 significantly decreased MMP-3 protein expression in U87MG and SNB19 glioma cells (Figure 5B). Furthermore, knockdown of CLC-3 significantly decreased MMP-9 mRNA and protein expression in U87MG and SNB19 glioma cells (Figure 5C, 5D).

## DISCUSSION

Overexpression of CLC-3 has been proved to be involved in carcinogenesis and tumor progression in variety of types of tumors [5]. This study provides the first evidence to our knowledge that CLC-3 is overexpressed in glioma tissues and positively correlated with WHO histological grade. Patients with high CLC-3 expression had an overall shorter survival time, whereas patients with low expression of CLC-3 had a better survival time. Knockdown of CLC-3 with ShCLC-3 adenovirus inhibits transcriptional activity of NF-κB, reduces MMP-3 and MMP-9 expression and decreases glioma cell migration and invasion. The results suggest that CLC-3 would be a promising target for therapeutic intervention of human gliomas.

It is believed that CLC-3 chloride channels play a critical role in cell migration and invasion by regulating cell volume [5]. CLC-3 chloride channels mediate the secretion of Cl^−^ along with obligated water to accomplish cell volumetric alteration and shape adaptation, which facilitate glioma cell invasion into narrow and tortuous extracellular spaces. In situ image showed the invading glioma cells adhere to blood vessels often assumed an elongated spindle-shaped morphology [24]. It was found that CLC-3 protein was upregulated in cell membrane both in glioma cell lines and glioma patient samples [20]. Specifically, CLC-3 chloride channels colocalized with Ca^2+^-activated K^+^ (BK) channels to the invadipodia of glioma cells [4]. Ca^2+^-activated BK channels serve as the principal pathway for K^+^ efflux from glioma cells, and the activation of Ca^2+^-activated BK channels is required for glioma migration [25]. This colocalization indicated glioma cells orchestrated ion channels to dynamically regulate the leading process, which facilitated cell invasion. It is commonly seen in the clinic that peritumoral brain edema increase obviously along with the increase of the pathologic grade of glioma [26]. It is found that peritumoral brain edema is significant contributor to morbidity and mortality from glioma [27]. The pathologic consequence and underlying mechanism remain to be understood. Activation of CLC-3 chloride channels to promote cell invasion might play a functional role.

This study finds that knockdown of CLC-3 inhibits p65 nuclear translocation, decreases transcriptional activity of NF-κB, and reduced MMP-3/9 expression, indicating that during metastasis, glioma cells not only passively regulate cellular volume and morphology to navigate the constrained space via CLC-3 chloride channel-mediated Cl^−^ efflux, but also actively create an extracellular microenviroment that favors invasion through CLC-3 channels. The upregulated CLC-3 in gliomas promotes NF-κB transactivity to regulate intracellular transcriptomic plasticity, alters matrix metalloproteinase such as MMP-3, MMP-9 expression, and actively modulates the extracellular environment. The mechanism of how CLC-3 regulates NF-κB signaling is unclear. Previous study in endothelial cells suggested CLC-3 -mediated Cl^−^ efflux decreased intracellular Cl^−^ concentration and promoted NF-κB activation [15]. In glioma cells, CLC-3 was found expressed on the plasma membrane as well as in the nucleus, interacting with nuclear α-tubulin [16]. Whether the nuclear CLC-3 affects NF-κB activation remains unknown. Notably, glioma cells have a basal Cl^−^ current at rest, leading to Cl^−^ efflux [28]. Interestingly, it was found that NF-κB, which is normally quiescent and dependent on specific molecular signals in many cells, is constitutively activated in gliomas, and the expression of NF-κB -drived genes are increased, which are inversely correlated with patient prognosis [11]. The precise molecular mechanism underlying CLC-3- mediated aberrant activation of NF-κB signaling needs further investigation.

NF-κ B is a pleiotropic transcription factor which plays important roles in regulation of many immune, inflammatory and carcinogenic responses. Targeting NF-κ B in cancer treatment can produce side effects such as immunotoxicity [11]. TM-601, a small 36-amino-acid peptide served as CLC-3 channel inhibitor [24, 29], is found to selectively bind to glioma cells but not normal brain parenchyma. Treatment of malignant gliomas with TM-601 is well tolerated and promising, and is currently in phase II clinical trials [30]. Given functional role of CLC-3 in regulation of NF-κ B signaling, TM-601might present a useful alternative to target aberrant NF-κ B activation in other pathological contexts.

## MATERIALS AND METHODS

### Tissue specimens

A total of 89 paraffin-embedded glioma specimens, obtained from astrocytoma patients (WHO grade I tumours, 9; grade II, 25; grade III, 20; and grade IV, 35) were collected for this study; All these patients had no chemotherapy, or radiation therapy before underwent initial surgical resection. They had been histopathologically and clinically diagnosed at the Sun Yat-sen University-Affiliated First Hospital by at least two independent pathologists separately. Six normal brain tissues were obtained by donation from individuals who died in traffic accident and confirmed to be free of any prior pathologically detectable conditions by at least two independent forensic pathologists separately at the center for medicolegal expertise of Sun Yat-sen University. Prior patient's consents and approvals from the Institutional Research Ethics Committee were obtained.

### Cell culture

Glioma cell lines, U87MG, SNB19, LN229 and U373MG (American type culture collection) were maintained in Dulbecco's modified Eagle's medium (Invitrogen) supplemented with 5% FBS (HyClone).

### Recombinant adenovirus

Short hairpin RNA targeted human clc-3 sequence (NM_001243374.1) is 5′-CCACGACUGGUUUAUCUUUCUCAAGAGAAAAGAUAAACCAGUCGUGG-3. Control scrambled sequence is 5′-UUCUCCGAACGUGUCACGUUUCAAGAGAACGUGACACGUUCGGAGAA-3′ High titers of recombinant adenoviruses were generated to knockdown CLC-3 by using the AdEasy system (MP Biomedicals Inc.) as described previously [31, 32].

### Preparation of cytopasmic and nuclear extracts

Nuclear and cytoplasmic proteins were extracted with NE-PER® Nuclear and Cytoplasmic Extraction Reagents according to the manufacturer's instructions (Thermo Scientific).

### Immunohistochemistry analysis

Immunohistochemistry (IHC) staining was performed using a standard streptavidinbiotin-peroxidase complex method as described previously [6]. The CLC-3 protein expression levels were evaluated by microscopic examination of stained tissue slides. CLC-3 expression level was determined by integrating the percentage of positive tumor cells and the intensity of positive staining. The intensity of staining was scored as follows: negative(score 0), bordering (score 1), weak (score 2), moderate (score 3), and strong (score 4). We scored the staining extent according to the percentage of positive stained tumor cells in the field: negative (score 0), 0–25% (score 1), 26–50% (score 2), 51–75% (score 3), and 76–100% (score 4). The product of the intensity and extent score was considered as the overall IHC score (values: from 0 to 16). The staining was observed and assessed by two independent pathologists.

### Western blotting

Western blotting were performed as described previously [6], using anti-CLC-3, anti-MMP-9, anti- Lamin A (Abcam), anti-p65, anti-β-tubulin (Cell signaling) antibodies.

### Quantitative real-time polymerase chain reaction (qRT-PCR)

The expression of *MMP3 and MMP9 mRNA* were determined by qRT-PCR using Power SYBR green PCR master mix (Applied Biosystems) as described previously [6] and primers were shown in Supplementary Table 1.

### Transwell migration assay and invasion assay

Cells (2 × 10^4^) in serum free medium were added on the top side of the polycarbonate Transwell filter without (for Transwell migration assay) or with Matrigel coating (for Transwell matrix penetration assay) in the upper chamber of the BioCoat^TM^ Invasion Chambers (BD) and incubated at 37°C for 20 hrs (Silencing CLC-3 by ShCLC-3 adenovirus did not significantly reduce the glioma cell numbers at 20 hrs as confirmed by prior MTT assay, Supplementary Figure 1), followed by removal of cells inside the upper chamber with cotton swabs. Migrated and invaded cells on the membrane bottom surface were fixed with methanol, stained with 1% crystal violet and counted using a light microscope in 5 random visual fields at the magnification of 100X.

### Cl^−^ currents recording

Membrane whole-cell Cl^−^ currents were recorded with an Axopatch 200B Amplifier (Axon Instrument) as previously described [6, 22]. Briefly, the currents were elicited with voltage steps from −100mV to +120mV in +20mV increment for 400ms with an interval of 5s from a holding potential of −40mV. Currents were sampled at 5 kHz using pCLAMP8.0 software (Axon Instruments) and filtered at 2 kHz. To minimize the changes of liquid junction potentials, a 3 mM KCl-agar salt bridge between the bath and the Ag-AgCl reference electrode was used. All experiments were performed at room temperature (25°C).

### Luciferase assay

pNF-κB-luc containing the minimal promoter with multiple tandem NF-κB binding sites and the control-luciferase plasmids (Clontech) were used to quantitatively examine NF-κB activity according to the manufacturer's instructions. Glioma Cells (3.5 × 10^4^) were seeded in triplicates in 48-well plates and allowed to settle for 24 h. One hundred nanograms of pNF-κB-luc plasmid, or the control plasmid, plus 1 ng of pRL-TK renilla plasmid (Promega), were transfected into glioma cells using the Lipofectamine^TM^ reagent (Invitrogen) according to the manufacturer's recommendation. Luciferase and renilla signals were measured 48 h after transfection using the Dual Luciferase Reporter Assay Kit (Promega).

### Enzyme-linked immunosorbent assay (ELISA)

The concentrations of MMP-3 and MMP-9 in the cell conditioned medium were determined by sandwich ELISA using MMP-3 and MMP-9 ELISA Kit (R&D Systems) according to the manufacturer's instructions.

### Statistical analysis

All experiments represent at least six independent replications. The chi-square test was used to analyze the relationship between CLC-3 expression and clinicopathological characteristics. Survival curves were plotted by the Kaplan–Meier method and compared using the log-rank test. Survival data were evaluated using univariate and multivariate Cox regression analyses. Statistical analyses were carried out using the SPSS13.0, with values of P ≤ 0.05 were considered significant.

An expanded Materials and Methods section is in the supplementary materials.

## SUPPLEMENTARY MATERIALS FIGURES AND TABLES



## Abbreviations

NF-κB: nuclear factor-κB
IHC: immunohisto-chemistry
OS: overall survival
ELISA: enzyme-linked immunosorbent assay
qRT-PCR: quantitative real-time polymerase chain reaction
MMP: matrix metalloproteinase
