# Supplementary material for: Suppression of CLC-3 chloride channel reduces the aggressiveness of glioma through inhibiting nuclear factor-κB pathway
Source: Oncotarget. 2017 Jul 8;8(38):63788–98. doi: 10.18632/oncotarget.19093 (PMC5609961; doi:10.18632/oncotarget.19093)
Supplement: Supplementary file 1 [file oncotarget-08-63788-s001.pdf]

Suppression of CLC-3 chloride channel reduces the aggressiveness of glioma through inhibiting nuclear factor-κB pathway

SUPPLEMENTARY MATERIALS

Cl- currents recording

Perforated whole-cell Cl- currents were recorded with an Axopatch 200B Amplifier (Axon Instrument). Pipettes were made from borosilicate glass using a two-stage puller (P-97, Sutter) and had the resistances of 3–5MΩ when the pipettes were filled with the pipette solution. Data were sampled at 20 kHz, low-pass filtered at 6.7 kHz. Series resistance was compensated by 90 %. Cells in a 500 µl chamber were continuously superfused at the rate of 2 ml per min. The isotonic solution (300 mosmol/kg·H2O) contained (in mM): 107 N-methyl-D-glucamine chloride (NMDG-Cl), 1.5 MgCl2, 2.5 MnCl2, 0.5 CdCl2, 0.05 GdCl3, 10 glucose,10 HEPES, and 70 D-mannitol, pH 7.4 with NMDG. The hypotonic solution (230 mosmol/kg·H2O) was made by omitting D-manitol from the isotonic solution. The osmolarity of the solutions was measured by a freezing point depression osmometer (OSMOMAT030, Germany). Recordings were started 5 min after the establishment of the whole-cell configuration to allow for the equilibration of the pipette solution with cell interior. The currents were elicited with voltage steps from -100mV to +120mV in +20mV increment for 400ms with an interval of 5s from a holding potential of -40mV. Currents were sampled at 5 kHz using pCLAMP8.0 software (Axon Instruments) and filtered at 2 kHz. A 3 mM KCl-agar salt bridge between the bath and the Ag-AgCl reference electrode was adopted to minimize the changes of liquid junction potentials. All experiments were performed at room temperature (25°C).

Western blotting

Cells were lysed for 30min in lysis buffer (in mM: 50 Tris-HCl, 150 NaCl, NaN3 0.02%, Nonidet P-40 1%, sodium dodecyl sulfate 0.1%, sodium deoxycholate 0.5% and 1% protease inhibitor cocktail). After centrifugation at 12,000 9 g for 30 min (4°C), the supernatant was collected for protein determination using Bradford reagent (Bio-Rad Laboratories). The samples were resolved by 8% sodium dodecyl sulfate–polyacrylamide gel electrophoresis, and separated proteins were transferred to polyvinylidene difluoride membranes (Bio-Rad Laboratories) and identified by immunoblotting. Primary antibodies (anti-CLC-3, anti-MMP-9, and anti- Lamin A antibodies were from Abcam; anti-p65 and anti-β-tubulin from Cell signaling) were diluted according to manufacturer's instruction, while secondary antibodies including horseradish peroxidase (HRP)-conjugated anti-rabbit and anti-mouse antibodies were obtained from cell signaling. Samples containing 40 µg of protein were subjected to SDS-PAGE and transferred to PVDF membranes. Membranes were blocked at room temperature for 1 h in PBST (in mM: 130 NaCl , 2.5 KCl, 10 Na2HPO4, 1.5 KH2PO4, 0.1% Tween-20, and 5% bovine serum albumin, pH 7.4), incubated with primary antibodies overnight at 4°C and then with the appropriate secondary peroxidase-conjugated antibodies 1 h at room temperature. Blots were developed using a chemiluminescence system and then were visualized by exposure to Kodak X-ray film. Density of target bands was accurately determined using a computer aided 1-D gel analysis system. Data were normalized with control.

MTT assay

Cell growth was determined by 3-[4,5-dimethylthiazol-2-yl]-2,5- diphenyl tetrazolium bromide (MTT) incorporation. Cells transfected with ShCLC-3 adenovirus or SCR adenovirus were seeded in 96-well culture plates in serum free medium for 20h. MTT assay was performed thereafter. Briefly, add MTT Solution in an amount equal to 10% of the culture volume, then return cultures to incubator and incubate for 3–4 h at 37°C. After incubation period, remove the culture fluid. Add MTT Solvent in an amount equal to the original culture volume. Plates should be read within 1 h after adding MTT Solvent. Spectrophotometrically measure the absorbance at a wavelength of 570 nm. Subtract background absorbance measured at 690 nm.

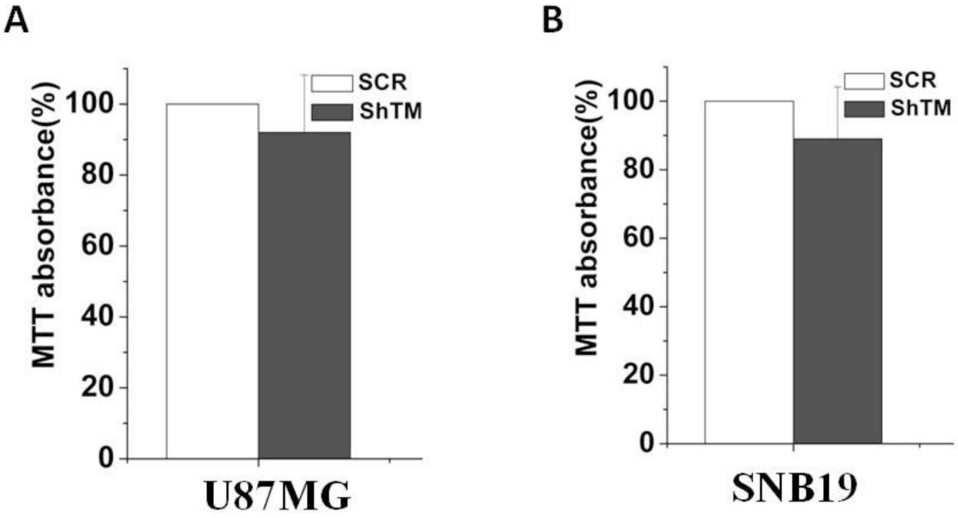

Supplementary Figure 1: Knockdown of CLC-3 did not significantly affect growth of U87MG **(A)** and SNB19 **(B)** glioma cells cultured in serum free medium for 20h as indicated by MTT assay.

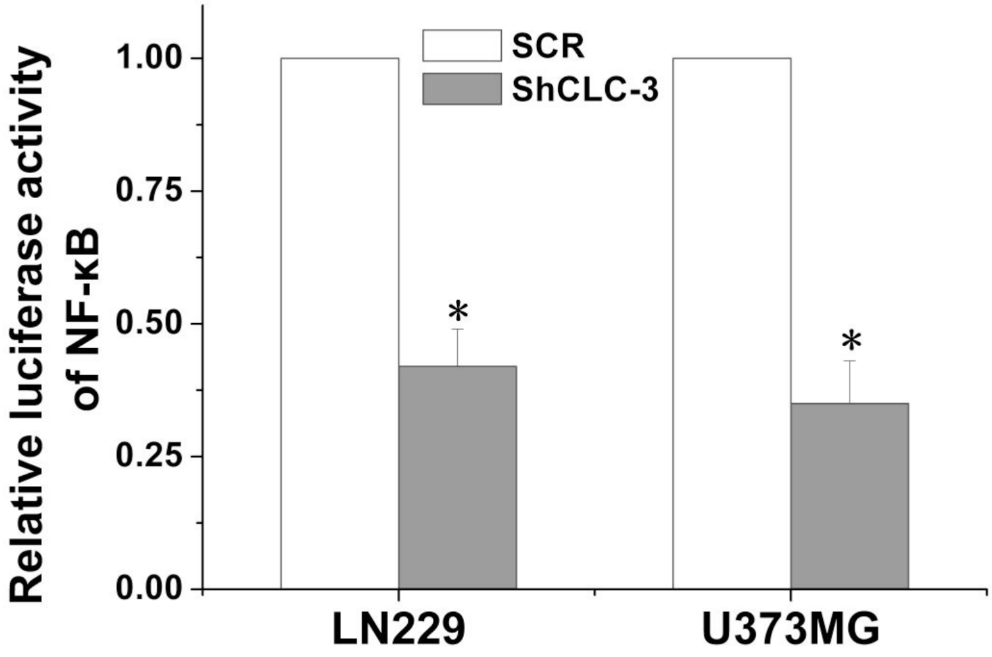

Supplementary Figure 2: Knockdown of CLC-3 significantly decreased the transcriptional activity of NF-κB as indicated by the luciferase reporter activity assay in LN229 and U373MG cells (n = 6, *p < 0.05 vs. SCR group).

Supplementary Table 1: Primers used for real-time PCR

Gene and primer		Sequences(5′to 3′)
NM_002422.3	mmp3_F	AGTGGAGGAAAACCCACCTT
	mmp3_R	CCAGGTCCATCAAAAGGGTA
NM_004994.2	mmp9_F	ACGACGTCTTCCAGTACCGA
	mmp9_R	TTGGTCCACCTGGTTCAACT
NM_002046.3	GAPDH_F	GCACCGTCAAGGCTGAGAAC
	GAPDH_R	TGGTGAAGACGCCAGTGGA

Supplementary Table 2: Correlation of CLC-3 expression with clinicopathological parameters

Variable	All cases	CLC-3 expression		P value
		High	Low	
Gender				
Male	62	43	19	0.150
Female	27	14	13	
Age at surgery				
<35b	47	35	12	0.329
≥35	42	28	14	
WHO grade				
I	9	3	6	**0.002**
II	25	11	14	
III	20	13	7	
IV	35	30	5	

aChi square test; bmedian age
